# State-of-the-art in the pneumococcal field: Proceedings of the 11^th^ International Symposium on Pneumococci and Pneumococcal Diseases (ISPPD-11)

**DOI:** 10.1186/s41479-019-0064-y

**Published:** 2020-02-05

**Authors:** Brenda Anna Kwambana-Adams, E. Kim Mulholland, Catherine Satzke, ISPPD group, Brenda Anna Kwambana-Adams, E. Kim Mulholland, Catherine Satzke, Heidi Smith-Vaughan, Angela Brueggemann, Cynthia Whitney, Lea-Ann Kirkham, Raquel Sá-Leão, Jorge Vidal, Hamish Graham, David Murdoch, Glaucia Paranhos-Baccala, David Goldblatt, William S. Pomat, Emma Best, Peter McIntyre, Jodie McVernon, Daniel Weinberger, Eileen Dunne, J. Anthony Scott, Allan W. Cripps, Grant Mackenzie, Shabir Madhi, Paul Torzillo, Steve Graham, Cissy Kartasasmita, Juliet O. Awori, Amber Smith, Markus Hilty, Chris Blyth, Tamara Pilishvili, Laura Hammitt, Ross Andrews, Kristy Crooks, William P. Hanage, Odilia Wijburg, Susan Morpeth, Neil French, Allen Cheng, Claudia Trappetti, Elaine Tuomanen, Jason Rosch, Narendra Arora, Gail Rodgers, Lay Myint Yoshida, Peter Richmond, Paul Licciardi, Daniela M. Ferreira

**Affiliations:** 1 NIHR Global Health Research Unit on Mucosal Pathogens, Division of Infection and Immunity, University College London, London, UK; 2 Murdoch Children’s Research Institute, Parkville, VIC Australia; 3 Department of Paediatrics, The University of Melbourne, Parkville, VIC Australia; 4 London School of Hygiene and Tropical Medicine, London, WC1H UK; 5 Department of Microbiology and Immunology, The University of Melbourne at the Peter Doherty Institute for Infection and Immunity, Melbourne, Victoria Australia

**Keywords:** *Streptococcus pneumoniae*, Pneumonia, Meningitis, PCV, Replacement, ISPPD, Asia, Indigenous

## Abstract

The International Symposium on Pneumococci and Pneumococcal Diseases (ISPPD) is the premier global scientific symposium dedicated to the exchange, advancement and dissemination of the latest research on the pneumococcus, one of the world’s deadliest bacterial pathogens. Since the first ISPPD was held in 1998, substantial progress has been made to control pneumococcal disease, for instance, more than half of surviving infants (78.6 million) from 143 countries now have access to the life-saving pneumococcal conjugate vaccine (PCV). The 11th ISPPD (ISPPD-11) was held in Melbourne, Australia in April 2018 and the proceedings of the symposium are captured in this report.

Twenty years on from the first ISPPD, there remain many challenges and unanswered questions such as the continued disparity in disease incidence in Indigenous populations, the slow roll-out of PCV in some regions such as Asia, the persisting burden of disease in adults, serotype replacement and diagnosis of pneumococcal pneumonia. ISPPD-11 also put the spotlight on cutting-edge science including metagenomic, transcriptomic, microscopy, medical imaging and mathematical modelling approaches. ISPPD-11 was highly diverse, bringing together 1184 delegates from 86 countries, representing various fields including academia, primary healthcare, pharmaceuticals, biotechnology, policymakers and public health.

## Background

*Streptococcus pneumoniae* (the pneumococcus) is a versatile pathogen that causes mucosal infections such as otitis media as well as life-threatening infections including pneumonia and meningitis [1].

The first International Symposium on Pneumococci and Pneumococcal Diseases (ISPPD-1) was convened in 1998, a time when there was no licensed pneumococcal vaccine for infants, who bear the brunt of invasive pneumococcal disease (IPD). The 7-valent pneumococcal conjugate vaccine (PCV) was licensed and introduced in 2000, markedly reducing the incidence of IPD caused by vaccine serotypes [2–4]. In 2000, an estimated 14.5 million total illnesses and 735,000 childhood deaths were attributed to the pneumococcus [5], which placed this Gram positive bacterium among the most important killers of children under 5 years of age. As of 2018, the 10-valent (PCV10) and 13-valent (PCV13) formulations of PCV had replaced the 7-valent version in 2011 and have now been introduced in 143 countries globally, with 58% (78.6 million) of infants having access to these life-saving vaccines [6]. Between 2000 and 2015, there was an estimated 51% decline in pneumococcal deaths among children less than 5 years old [7].

Although substantial progress has been made to control pneumococcal disease, there remain many challenges and unanswered questions to be addressed. These were the foci of the 11th ISPPD (ISPPD-11), that was held in Melbourne, Australia from April 15^th^ to 19^th^ 2018. Areas of focus were; access to PCVs, continued disparity in disease incidence in Indigenous populations, burden of disease in adults and serotype replacement. Appropriately, the program for ISPPD-11 put the spotlight on Indigenous communities in the region and around the globe who still face unacceptably high rates of IPD, other pneumococcal diseases and their sequelae. For example, the slow roll-out of PCV in some regions such as Asia and the high burden of IPD in adults where PCV impact has been modest in some regions [8]. ISPPD-11 also brought the very latest scientific innovations in the field including in metagenomics, transcriptomics, microscopy, medical imaging and mathematical modelling. The abstracts are available online at https://isppd.kenes.com/2018/Documents/ISPPD-11%20Abstract%20Book.pdf.

ISPPD-11 brought together 1184 delegates from 86 countries, representing various fields including academia, primary healthcare, pharmaceuticals, biotechnology, policymakers and public health. A record high 184 delegates from 51 countries were awarded travel fellowships to support their attendance, representing 16% of the delegates at ISPPD-11.

Below we describe the key highlights from the plenary and parallel sessions.

### Pneumococcal disease: the young, the old and the vulnerable

#### Infants

Infancy is a time of vulnerability for pneumococcal acquisition, carriage and disease. Presentations on infant disease focused on how early life acquisition of pneumococci, and other factors such as malnutrition, increase susceptibility to disease and risk of death. Another area of emphasis was the optimal dosing schedules to sustain PCV immunization programs, with emphasis on GAVI graduating countries.

Innovative mathematical modelling demonstrated how early infections increased otitis media “proneness” [9]. Similarly, lower respiratory tract infections (LRI) with onset early in life were associated with progression to suppurative lung disease (bronchiectasis). It is reassuring, however, that PCV programs lessen otitis media “proneness” and delay the timing of the first bacterial pneumonia hospitalization in infancy among Australian Indigenous populations, holding promise for improved child health [10].

Other clinical and socio-demographic factors may influence IPD and IPD-related mortality in early life. A landmark study involving 16,000 Kenyan children with non-severe pneumonia showed that the most important risk factors of death were moderate malnutrition (weight for age) and clinically severe pallor [11].

The World Health Organization (WHO) recommends three primary PCV doses given at 6, 10 and 14 weeks or 2, 4 and 6 months of age. Alternatively, two primary doses plus a booster given between the age of 9 and 15 months is also recommended [12]. There is interest in understanding the optimal number of PCV doses and the timing of the doses [13] while balancing cost and program sustainability. The potential benefits of a booster-containing regimen are being evaluated in many studies in low- and middle-income countries (LMIC) and results from Nepal and Vietnam are highlighted here. A study of 2 different 2 + 1 PCV10 schedules in Nepal (either 6, 14 weeks plus a booster prior to 1 year or 6, 10 weeks plus a booster dose given before 1 year of age, produced comparable antibody responses in Nepali infants following the booster, although antibody levels after the primary series were lower in the 6 + 10 week age groups [14]. Comparison of a PCV10 3-dose primary series (2, 3, 4 months) was found to produce slightly higher serotype specific antibody concentrations than a 2-dose primary schedule (2 and 4 months). However, following a booster given at 9 months, the 2 + 1 schedule produced significantly higher antibody levels, potentially demonstrating an advantage of the 2 + 1 schedule in this setting [15]. When comparing PCV13 and PCV10 using the 2 + 1 schedule, PCV13 produced higher antibody titers than PCV10 for most serotypes post-primary series, while similar levels were seen for both vaccines at 9 months pre-booster and following the booster [15].

In Malawi, administration of PCV13 as a 3 + 0 schedule demonstrated good infant protection against IPD, with herd (indirect) effects demonstrated in older children [16]. Key to the optimal dosing issue is the need for high coverage. For example, Nigeria, Africa’s most populous country, has PCV coverage below 30% despite the pneumococcus being the most common cause of meningitis [17]. The data presented suggested that with adequate coverage, PCV10 could have prevented 50% of cases of pneumococcal meningitis in Nigerian children under five years reported between 2010 and 2016 [17]. An action plan is needed to ensure high vaccine coverage in high-mortality regions.

Although the infant vaccination programs have been largely successful, protection of newborns against pneumococcal disease remains a challenge in some regions. Although the mechanisms are poorly understood, experiments in neonatal mice suggest that T-helper cell activation may be impaired in newborns [18]. Maternal vaccination may provide protection during the neonatal period, a strategy that has been implemented successfully against diseases such as pertussis and tetanus [19]. However, there are concerns that maternal antibodies may interfere with subsequent immune responses following administration of PCV among infants.

Definitive data on the safety and effectiveness of PCV maternal vaccination is required as is the need for enhanced understanding of the burden of disease in very young infants and key differences to that of older infants. As the jury is still out on the optimal PCV dosing and scheduling, it is important to note that there may not be a “one-size fits all” strategy for all countries and populations.

#### Adults

Among the elderly, community acquired pneumonia (CAP) is the most common type of pneumococcal disease. The dominant presentation of pneumococcal CAP is non-bacteraemic/non-invasive disease while invasive infections involving normally sterile sites occur in a quarter of cases [20]. Vaccines which contain purified capsular polysaccharides (PPVs) are effective against invasive disease in adults but their efficacy against non-invasive pneumonia remains controversial [21, 22]. It is also unclear how PPV protection against disease and carriage wanes over time in adults [22]. At ISPPD-11, there was a focus on protection of adults and innovations for timely diagnosis of CAP.

Japan has experienced an increase in uptake of the 23-valent polysaccharide vaccine (23vPPV) among the elderly following the 2011 tsunami. The free pneumococcal vaccinations offered to tsunami victims >65 years may have contributed to the increased uptake [23]. Suzuki and colleagues [24] found that effectiveness of 23vPPV against pneumococcal pneumonia was 27% but wanes over time and with increasing age at vaccination. Macintyre and colleagues conducted a randomized control trial (RCT) among hospitalized elderly Australians who were recruited to receive either 23vPPV alone or PCV7 and 23vPPV [25]. They found substantial waning of immunity between 12 and 60 months post vaccination for all serotypes using the opsonophagocytic assay (OPA). The clinical implications of this waning immunity remain unclear.

An alternative to 23vPPV is PCV vaccination among adults. The Community-acquired Pneumonia Immunization Trial in Adults (CAPITA) [26] study conducted in the Netherlands showed that the vaccine efficacy of PCV13 among adults >65 years was 45.6% (95% CI: 21.8–62.5) for the first episode of vaccine-type CAP. The efficacy for non-invasive CAP was much lower 45.0% (95%  CI: 14.2–65.3) compared to invasive CAP 75.0% (95% CI: 41.4–90.8). At ISPPD-11, Gessner and colleagues [27] presented findings from a post hoc analysis of The CAPITA study. Vaccine efficacy for all hospitalized pneumonia was comparable for clinical pneumonia 8.1% (95% CI: − 0.6-16.1) and adjudicated (clinical plus radiological) pneumonia 6.7% (95% CI: 4.1–16.23). Vaccine efficacy was highest against hospital episodes for vaccine type IPD 75.8% (95% CI: 47.6–98.8) and IPD 49.3% (95% CI: 23.2–66.5). Together, these findings support that PCV13 is effective in preventing both invasive and non-invasive vaccine-type CAP. Questions remain regarding the cost effectiveness of rolling out PCV13 for adults with existing immunization programs [28]. The final decisions will largely depend on the herd immunity conferred by infant immunization programs and the lower cost of 23vPPV compared to PCV13. For example, in 2019, the Advisory Committee on Immunization Practices (ACIP) downgraded the universal recommendation for PCV13 use for adults >65years and decisions to administer PCV13 will now be based on shared clinical decision-making. The updated recommendation was supported by the finding that herd immunity from PCV13 infant immunizations made a more significant impact than vaccinating adults in the US [29].

Although a significant proportion of pneumococcal pneumonia remains undiagnosed, surveillance for invasive pneumococcal disease provides indirect data on serotype distribution and the changing incidence of non-invasive disease. Data on CAP incidence among adults in Sub-Saharan Africa are scant, but the incidence of pneumococcal pneumonia is known to be very high in people with human immunodeficiency virus (HIV) infection [30]. In South Africa, the highest incidence of IPD was reported in adults 25–44 years, the age group with the highest HIV prevalence [31]. Seven years after the introduction of PCV in South Africa, IPD rates had declined by 45% (95% CI: 43 − 48%) in adults >25 years of age, despite some replacement disease. In contrast with many high income countries, routine pediatric vaccination in South Africa appears to have had little indirect impact on IPD in older adults ≥65 years of age.

The global burden of IPD will likely grow with the increasing ageing population and replacement of vaccine-serotypes [32]. There is a need to focus research on the most effective vaccine strategies to protect the elderly from IPD and pneumonia. In addition to HIV prevention/treatment, other measures to reduce IPD include approaches to reduce uptake of cigarette smoking and indoor air pollution from cooking, as well as optimize herd protection from childhood pneumococcal vaccination.

#### Indigenous populations world-wide

Issues of Indigenous health took center stage at ISPPD-11. Indigenous communities deserve the spotlight for their role in research that led to watershed advances in vaccinology and pneumococcal epidemiology. Despite the high effectiveness of PCVs in Indigenous populations, particularly in reducing the burden of vaccine-type IPD, data presented from Australia and the United States of America (USA) highlight the ongoing pneumococcal disease disparity between Indigenous populations and the general population.

Data from Australia’s National Notifiable Disease Surveillance System showed that IPD remains a significant health burden in Australian Indigenous populations [33]. The disparity between Indigenous and non-Indigenous Australians is actually increasing and is most pronounced in young adults. Similarly, in the USA, the residual burden of IPD on the Navajo Nation is higher than the general population [34]. These differences in populations must be taken into account when assessing research questions and policy issues, such as optimal dosing schedules [35].

Additionally, other aspects pertinent to Indigenous populations, especially in Aboriginal and Torres Strait Islander communities across Australia, were highlighted by Australia’s first Indigenous Ear Nose and Throat (ENT) surgeon, Dr. Kelvin Kong. He challenged delegates at ISPPD-11 to think beyond the pneumococcus and to look beyond vaccines [36], emphasizing the unacceptable toll that otitis media – and its sequelae – continues to exert in Aboriginal and Torres Strait Islander communities across Australia, resulting in social isolation, poor educational outcomes and lost opportunities. He called out the pervasive racism that continues to create barriers to care-seeking, preventing appropriate recognition and treatment of disease, and called for community-driven, culturally-appropriate, locally-delivered solutions that reflect the social reality of Aboriginal and Torres Strait Islander communities.

Five clear priorities were identified for the reduction in disease in Indigenous populations: 1) maximizing vaccine coverage, 2) ensuring policy-relevant data collection, 3) optimizing dosing products and schedules, 4) evaluating new vaccines with broader serotype protection, and 5) addressing the social determinants of health that underlie the high burden of pneumococcal disease.

#### Epidemics, outbreaks and emergency settings

Optimal vaccination strategies are a priority for humanitarian emergencies, which affect 150–200 million refugees and internally displaced persons annually [37]. People living in overcrowded places such as refugee camps and other poor living conditions are at risk of pneumococcal outbreaks [38–42]. In all refugee communities, pneumonia continues to be an important cause of illness and death. The Humanitarian Mechanism, launched in 2017, represents an important initiative providing access to PCVs and potentially other vaccines at GAVI prices [43].

The message was clear at ISPPD-11, we need vaccination programs that are feasible, provide rapid protection, maintain immunity over a protracted phase, are cost-effective, and minimize opportunity costs through integration with other health services [44]. Mathematical modelling approaches can be applied to determine the optimal strategy, cost-effectiveness, and need for use of PCV in humanitarian settings [45].

In Sub-Saharan Africa’s “meningitis belt” which spans from Senegal to Ethiopia, there are seasonal meningitis outbreaks, primarily caused by *Neisseria meningitidis* but including many cases caused by pneumococci. Outbreaks of pneumococcal meningitis continue to pose a significant public health threat along Africa’s meningitis belt. Kwambana-Adams outlined how pneumococcal meningitis outbreaks peak during the dry and dusty season, and are characterized by high rates of mortality and serious sequelae in survivors [46]. Serotype 1 dominates, but meningitis cases are also being caused by other pneumococcal serotypes. There has been a shift in the age distribution of meningitis cases following PCV introduction, from primarily children under 5 years to a predominance in older children and adults [46, 47]. Notably, in the Gambia the serotype 1 carriage reservoir is likely to be in the 5–14 year age group [48]. Other pneumococcal IPD outbreaks were reported among the homeless in Canada, attributed to two serotype 4 genotypes and in Israel related to a unique clone of serotype 2 [49].

Infant vaccination programs alone may not be sufficient to protect the vulnerable populations against IPD outbreaks across the globe. Optimal population-specific vaccination strategies targeting outbreak control are needed. Additionally, preparation for future outbreaks in Africa as well as the Asia-Pacific region requires strengthened surveillance [50].

#### Diagnosis and treatment

Timely diagnosis and the correct assessment of pneumococcal disease remains an immense challenge, even in high-resource settings [32], and may result in delayed or suboptimal treatment of patients and underestimation of the burden of disease. The new innovations presented at ISPPD-11, ranging from computer-aided platforms, low-cost pulse oximeters and diagnostics based on non-invasive clinical specimens, may revolutionize pneumococcal disease diagnostics. Tools that are accessible and can be used in low-resource settings are particularly promising.

Several presentations focused on the need for improved diagnosis of pneumonia in children, three of which are highlighted here. Discordance between the WHO clinical case definition of pneumonia and radiographic diagnosis (based on WHO criteria) was reported in a study from Nepal [51] where two-fifths of those with radiological pneumonia did not meet WHO clinical criteria, mostly due to a lack of tachypnoea. This discordance does not infer “incorrect” diagnosis, but rather highlights the limitations of different diagnostic standards and the importance of considering these limitations in research and practice. Mahomed and colleagues [52] presented a computer aided diagnostic algorithm for chest X-ray (CXR) interpretation for childhood pneumonia. Using expanded data from the Pneumonia Etiology Research for Child Health (PERCH) study South African site, the algorithm had 70% sensitivity and 72% specificity (compared to expert radiology opinion). Given the challenges of interpretation of CXRs in children this is promising, especially as there is capacity to further improve the diagnostic algorithm.

Improvement in measurement of hypoxemia in children was described by Boyd and colleagues [53]. They have developed a novel pediatric pulse oximetry probe and demonstrated both high quality and reuseability by both expert clinicians and health workers in Malawi, Bangladesh and the United Kingdom.

Non-invasive pneumococcal disease diagnostic tools for adults were presented. One study found that sputum PCR was more sensitive overall and better at detecting non-systemic pneumococcal pneumonia than urine antigen detection [54]. Although still requiring further validation, a new 24-valent serotype-specific urinary antigen test in adults enhanced diagnosis of pneumococcal pneumonia [55].

Minimally invasive post-mortem tissue sampling to determine cause of death, was also discussed at ISPPD-11, and may shed more light on the true burden of IPD in children in LMIC settings [56].

New treatment strategies for otitis media were also discussed. Using a chinchilla model, Sabharwal and colleagues [57] demonstrated that local administration of gemifloxacin-containing gel cleared pneumococcal acute otitis media in most (13/14) animals. They found that use of 4% ciprofloxacin and 1% gemifloxacin gel quickly sterilized the ear (90% within 5 h) with negligible systemic absorption and superior efficacy to no treatment or oral comparison treatments [57].

### Weighing the balance: vaccine impact and serotype replacement

Under the pressure of pneumococcal vaccines, pneumococcal epidemiology continues to evolve [58]. At ISPPD-11 the increasingly diverse impacts of PCVs on pneumococcal disease were presented. One of the most illustrative examples of impact differences is that of serotype replacement.

Lessa and colleagues [47] used inpatient data from 23 US states collected between 2005 and 2014 to determine the rates of all cause pneumonia (ACP), non-invasive lobar pneumonia (10th edition of the International Classification of Diseases (ICD10)-coded) and IPD. This study including 83.4 and 78.8 million hospitalizations pre and post PCV13 introduction respectively, showed reductions in lobar pneumonia hospitalizations. Approximately 23,674 (Credible Interval: 12,637 - 38,484) hospitalizations across all ages have been averted between 2010 and 2014, post PCV13 introduction on a 3 + 1 schedule. Declines in ACP were only observed among children <5 years but not among older children and adults. The decline in IPD hospitalizations were very modest across all age groups, the largest decrease of 1574 (Credible Interval: 907–3907) was found among those <18 years [47].

The Active Bacterial Core surveillance (ABCs) showed declines in overall and PCV13 IPD of 61% and 87% respectively among North American children <5 years following introduction of PCV13 [59]. Similar trends were observed among older age groups. Serotype replacement appears not to be a major problem in the USA, at this time [60].

Studies from other regions are sharply contrasted with the USA where pneumococcal disease caused by non-PCV13 serotypes is a major concern. European *Streptococcus pneumoniae* Invasive Disease Network (SpIDnet) includes 10 European countries with varying infant immunization schedules and PCV formulations. SpIDnet demonstrated an even more dramatic 84% increase in non-PCV serotypes following PCV implementation across Europe [61]. The United Kingdom (UK) has seen declines in IPD and PCV13-type IPD, offset by significant increases in non-PCV13 serotypes, particularly since 2013 [62]. The incidence of IPD caused by non-PCV13 serotype IPD has doubled, with serotypes 8, 12F and 9N responsible for > 40% of IPD cases in 2016/17. Nearly complete replacement disease has been observed in adults in the UK. Another interesting aspect of replacement in the UK is that early replacement was largely due to serotypes with a low case:carrier ratio prior to PCV13 introduction. However, the replacement under the pressure of PCV13 has been different, characterized more by serotypes with a greater case:carrier ratio. In South Africa, non-PCV13 serotypes increased by nearly a third, with serotypes 8 and 35B being the dominant serotypes following PCV13 implementation [27].

In a recent article, Lewnard and Hanage [63] explored potential factors that may account for the observed differences on the impact of PCV13 on disease caused by non-vaccine serotypes in the USA, UK and Europe. These include surveillance practices, population risk factors, transmission dynamics and pathogen evolution.

England and Wales have experienced a large increase in IPD in adults >45 years post PCV13 introduction in 2010, with 8, 9N, 12F and 15A being among the most notable non-vaccine serotypes [62].

Senghore and colleagues provided insights into vaccine serotype persistence in the Gambia, a setting with a 3 + 0 PCV13 schedule [64]. Transmission of serotype 1, the predominant serotype, was ongoing five years after PCV13 introduction coupled with clonal expansion of non-PCV13 serotypes.

Mungall and colleagues [65] questioned the dogma, through a systematic review of published literature, that the impact of PCV on IPD correlates with the impact on nasopharyngeal carriage. Although there was good correlation when all the serotypes were grouped into vaccine and non-vaccine types, the correlation was poor at the level of individual serotype, which warrants further investigation.

Overall, pneumococcal ecology continues to evolve with serotype replacement being observed in most, but not all, countries. Further investigation to understand the biological basis for serotype persistence and selection are required to inform development of future vaccines and vaccine programs.

### Vaccine studies from the Asia-Pacific

Despite having a large share of the global burden of pneumococcal disease, the Asia-Pacific region has lagged behind the rest of the world in the introduction of PCVs. Currently, countries that have introduced PCVs are evaluating impact, especially for pneumonia where vaccine type pneumococcal carriage among pneumonia admissions and/or among children in the community is being assessed.

In 2012, Fiji became one of the first countries in the region to introduce PCV. A comprehensive evaluation of PCV10 impact showed significant reductions in severe pneumonia (35%), hypoxic pneumonia (35%) and radiological pneumonia (28%) among children 2–23 months of age following PCV introduction with variable reductions in all cause pneumonia in the different age groups [66]. Nepal introduced PCV13 in a novel 2 + 1 schedule (6 weeks, 10 weeks and 9 months) in 2015 [67]. Significant decreases in vaccine-type carriage in pneumonia patients were observed in children <2 years of age.

Some countries in the region have elected to introduce PCV in a phased manner. In India, PCV13 introduction started in 2017 focusing on the five states with high child mortality (Uttar Pradesh, Bihar, Madhya Pradesh, Rajastan and Himachel Pradesh) reaching over 2 million children. Evaluation of IPD, pneumonia and NP carriage in pneumonia patients is ongoing. In Himachal, PCV13 is currently being used but in the coming years Indian manufacturers will likely enter the market with new PCV formulations [68]. In Mongolia, PCV13 introduction began in the capital, Ulaanbaatar, in 2016. The introduction was undertaken in a phased manner to facilitate evaluation of the impact on pneumonia admissions. As part of that impact evaluation, nasopharyngeal carriage is being monitored among children admitted to hospital with pneumonia [69].

Early evidence from countries leading the way in the region presented in this session provided much needed data to advocate for roll-out of PCVs throughout the region. In addition to introduction and measurement of impact, vaccine development is ongoing in India. Development of new PCVs by Indian manufacturers will increase the availability and reduce the cost of PCVs that may lead to increase usage of PCV in LMICs where PCV introduction is still lagging. Lower-cost PCVs may also ensure the sustainability of PCV among countries that graduate from GAVI support.

### Epidemiology and mathematical modelling

While PCVs have been in use for nearly twenty years, there is still much to learn about how pneumococci change in response to vaccination, and what the implications are of different vaccination strategies. There is also a need for non-inferior, low cost PCV formulations and/or alternate dose schedules that help sustainability for infant PCV programs. At ISPPD-11, presenters described classical observational epidemiology and mathematical modelling approaches to address these issues.

Croucher and colleagues [70] presented work that synthesizes pneumococcal population structure to identify drivers of vaccine-driven strain selection in disparate populations. This type of interdisciplinary approach represents the cutting-edge of the field and could be used to tailor optimal vaccine formulations in different populations.

Ojal and colleagues [71] used dynamic transmission models and economic analyses to show that continuing PCV will prevent 15,355 deaths in Kenya between 2022 and 2032 at a cost of USD 15.6 million annually. Thus, continued PCV use will be cost effective for the country. This study from Kenya is important for understanding the potential impacts of PCVs in low income settings and for decision-making. Sbarra and colleagues [72] presented a modeling framework for synthesizing estimates of PCV impact between countries, for evaluating variables associated with higher or lower impact, and for obtaining estimates of impact where the quality of local data is poor. This could aid in efforts to understand variability in PCV impact between settings and the evaluation of different dosing schedules.

To address the issue of optimal PCV dose scheduling, Choi and colleagues [73] used a dynamic transmission model to show that switching from a 2 + 1 to 1 + 1 PCV schedule in the UK would result in a modest increase in vaccine type carriage and IPD among infants. They proposed that the strong herd immunity established in this population would counter the effects of reduced doses. Although controversial, this work has important implications for ongoing policy discussions surrounding the intricate balance between cost savings and pneumococcal disease prevention.

Together, these studies demonstrate the importance of modelling in evaluating the impact of PCVs in a variety of settings and address ongoing challenges for policy, vaccine design and schedule optimization.

### New vaccines and new trials

Although having registered remarkable success, currently licensed pneumococcal conjugate and polysaccharide vaccines have limited valency, providing protection against a small proportion of nearly 100 known pneumococcal serotypes. PCV success is threatened by the ever-looming possibility of non-vaccine type replacement disease.

The progress on the development of PCVs with broader valency, PCV15 [74] and PCV24 [75] as well as a lower cost PCV10 from the Serum Institute of India [76], were discussed. Stacey and colleagues demonstrated that two different preparations of PCV15 both have safety profiles comparable with that of PCV13 [77]. Both PCV15 formulations (PCV13 serotypes plus 22F and 33F) induced serotype-specific antibody responses to all 15 serotypes in the vaccine, and these responses were non-inferior to PCV13 for common serotypes, measured both by serotype-specific opsonophagocytic activity (OPA) and IgG geometric mean titers (GMCs). The challenge remains as to how many additional serotypes can be added while maintaining safety and immunogenicity.

There is a pressing need to develop new vaccine strategies and the current focus is on protein and whole cell vaccines [78–81]. To date the results from clinical trials testing protein vaccines have not been promising, but there is still a need to develop an effective pneumococcal protein vaccine. A group based at the Papua New Guinea Medical Research Institute with Australian collaborators measured antibody responses to four pneumococcal protein vaccine candidates [82]. They found high levels of natural antibody against these proteins in early infancy, but the protective role of these antibodies is yet to be evaluated.

A discussion by a panel of experts highlighted several key challenges relating to vaccine development and evaluation. There is a need for protection against a broader breadth of serotypes, but interest in non-capsular pneumococcal vaccines has declined while evidence of the limitations of PCVs is growing stronger. The pathway to licensure is considered a key barrier to the development of new pneumococcal vaccines, particularly protein vaccines, as there are no known correlates of protection. Thus, engagement with regulators is needed, to develop new approaches for evaluating and licensing vaccines that account for potential ecological effects. Expanded valency PCVs may suffer from “GMC creep” whereby serotype-specific IgG responses may decrease as more serotypes are added to the vaccine. It is unknown when this becomes clinically significant. Additional questions arise given the fact that much of the pneumococcal disease burden resides in adults, but most work in vaccine development is focused on pediatric vaccines. Should we be making a different PCV for adults targeting serotypes causing IPD in adults? Should we be evaluating new vaccines initially in adults rather than children?

### Host and microbial interactions

#### Host-pathogen interactions

Fundamental to success of pneumococcal colonization and disease is the capacity of the organism to interact with epithelial and endothelial surfaces whilst evading host defenses [83]. The complex interplay between pathogen and host dictates tissue tropism and disease severity [84]. Speakers at ISPPD-11 demonstrated the utility of cutting-edge sequencing technologies and microscopy tools in advancing our understanding of the mechanisms underlying pneumococcal-host interactions.

The rapid transcriptomic reprogramming by both host and pathogen underscores the dynamic responses that drive successful pneumococcal colonization and facilitate invasive disease. Veening and colleagues [85] presented exciting data from dual RNA-seq performed in an innovative zebrafish embryo pneumococcal meningitis model. This study showed that *S. pneumoniae* strain D39 increased transcription of competence, pyrimidine and heat shock genes in response to interaction with brain tissue. Jhelum and colleagues [86] also demonstrated that pneumococci have mechanisms to package DNA-degrading enzymes into vesicles, which in turn facilitates the destruction of neutrophil extracellular traps.

Two-photon microscopy demonstrated that pneumococcal translocation into the central nervous system can occur through non-hematogenous routes involving the olfactory system in a mouse model [87]. This is further evidence of the remarkable ability of the pneumococcus to circumvent the blood-brain barrier to cause meningitis.

Through application of super-resolution microscopy of human meningitis samples, it was revealed that pneumococcal pili interacts with two blood-brain barrier endothelial receptors, polymeric immunoglobulin receptor (pIgR) and platelet endothelial cell adhesion molecule (PECAM-1), which in turn facilitate pathogen entry into the brain [88]. Targeting the intimate interactions between the pneumococcus and these receptors resulted in reduced bacterial translocation, demonstrating the potential for targeting such pathways therapeutically [89].

These findings underscore the multiple, potentially redundant, pathways that the pneumococcus can employ to invade the central nervous system. Mechanistic understanding of how the pneumococcus gains access to this highly privileged host niche can be leveraged for the development of interventions to prevent meningitis.

#### Immunology

In recent years, substantial progress has been made in our understanding of the immunological basis of protection against pneumococcal carriage. At ISPPD-11, we gained new insights into the complex interactions between host immunity and the pneumococcus, particularly at the mucosal surface. A number of speakers presented innovative approaches to enhance our knowledge of novel cellular immune responses associated with protection against pneumococcal carriage, impact of bacterial-viral inflammatory responses and new approaches to vaccination that could provide serotype-independent protection against pneumococcal disease.

A cutting-edge proteomic study showed that exposure to pneumococcus during the first two years of life elicited antibody responses to protein variants of PspA, PspC, ZmpA and ZmpB [90], and that vaccination with the whole cell vaccine (WCV) produced similarly consistent responses to functionally diverse proteins [91]. Intranasal vaccination in mice with conserved lipoproteins such as DacB, MetQ and PnrA was associated with reduced bacterial loads and increased nasal IL-17A levels. These studies offer hope that protein-based broad protection against the pneumococcus may be achievable in the future and support the further development of whole-cell and protein vaccines.

A human challenge model study showed enhanced activation of alveolar macrophages in individuals within 4 months of an episode of pneumococcal colonization. The clearance of pneumococci in the lungs may be modulated by the activity of alveolar macrophages and enhanced expression of IFN-ɤ and IL-4 [92].

A study of B-cell responses to pneumococcal serotypes following varied PCV schedules provided a new perspective on markers of long-term immunity. Examining the predictive power of this approach as a predictor of protection against carriage in Vietnam (where there is still no national PCV program) will be a report to look for at ISPPD-12 [15].

Effective protection of immunocompromised individuals against IPD remains a challenge as highlighted in previous sections. It was found that specific adaptive immune cells such as T follicular cells (Tfh) were important for antibody production in response to pneumococcal polysaccharide vaccination [93] but that this was impaired during HIV-infection, whereas an extra PCV13 booster could provide additional benefit among immunocompromised individuals [94].

In summary, significant advances continue to be made in our understanding of novel immune correlates of protection against pneumococcal carriage and disease. These findings will be critical in our efforts to develop broad-based protection against this highly complex pathogen.

#### Interaction between pneumococci and viruses

Pneumococci co-colonize or co-infect a host together with other pathogens, including viruses, bacteria, and/or helminths [95]. There are data on influenza-pneumococcal coinfection reviewed elsewhere [96], but epidemiological data indicate several other pathogens are co-detected in individuals with pneumococcal disease. How these alter pneumococcal prevalence, carriage, susceptibility, and disease course is not well understood. Speakers at ISPPD-11 highlighted the mixed etiologies of pneumococcal coinfection.

In Vietnam, among children under 15 years with acute respiratory illness, those colonized by a virus (respiratory syncytial virus (RSV), human-metapneumovirus, rhinovirus or adenovirus) were more likely to be co-colonized with the pneumococcus. Children with clinical pneumonia and RSV colonization appeared to have longer hospitalizations than those without RSV [97]. This finding is consistent with data reported from animal model studies of pneumococcal co-infections with RSV [98], *Trichuris muris* [99], and influenza virus [100]. Further investigation into RSV-pneumococcal coinfection showed that inflammatory markers and overall disease were also enhanced, but that epithelial integrity was maintained [98]. Like influenza-pneumococcal coinfections [96, 100–102] alveolar macrophages during *T. muris* infection exhibit reduced killing capacity [99]. In *T. muris*-pneumococcal coinfection, increased carriage and dissemination of pneumococci into the lungs was also observed [99]. Pneumococcal serotypes were found to be more diverse in mixed-infections [103].

The prevalence and mechanisms of multi-pathogen infections is beginning to be better understood. The complexity of these infections and the phenotypic changes that ensue in multi-pathogen pneumococcal infections is of high importance and should be further investigated. This may be an important factor explaining the link between pneumococcal disease incidence and microbial community development. The knowledge gained will undoubtedly aid the control of pneumococcal-related diseases in future.

### New frontiers in pneumococcal “omics”

#### Genomics and transmission

Exciting advances in genomic analyses exploring pneumococcal virulence, antibiotic resistance and population structure were discussed in depth at ISPPD-11.

Hiller demonstrated the significance of gene transfer in tissue tropism, focusing on a group of strains that form a distinct phyletic group within the pneumococcus that are well adapted to infect the conjunctiva [104]. The surface-exposed adhesin, SspB, is encoded exclusively by strains in this distinct phyletic group and promotes adherence to cultured cells from the ocular epithelium. Phylogenetic analysis of the *sspB* gene suggested that it was acquired by lateral gene transfer from *Streptococcus suis*.

Genomic approaches have also been used to address why and how antibiotic resistant and sensitive pneumococcal strains co-exist, which is hard to explain with simple models. Lehtinen and colleagues’ work demonstrated that duration of carriage can modulate the fitness effect of antibiotic resistance, whereby prolonged duration of carriage of a serotype increases the fitness advantage gained from resistance [105]. This is intuitively easy to understand, as the longer it takes for the pneumococcus to transmit, the greater the chance that the host will receive an antibiotic prescription for any infection. These findings suggest that any locus that increases the duration of carriage is linked with the evolution of resistance to antibiotics.

While capsular serotype is known to contribute to pneumococcal invasiveness, investigators at ISPPD-11 demonstrated that genetic lineage also contributes to invasiveness [106]. Clade specific clustering linked to either carriage or invasive disease was found among serotype 1 strains from West Africa [107]. A study employing novel genome-wide association study (GWAS) and joint sequencing approaches identified pneumococcal genes important in meningitis that are involved in competence, metabolic pathways and bacteriocins. Host variation in Coiled-Coil Domain Containing 33 (*CCDC33)* and serine/threonine kinase 32C (*STK32C*) were associated with susceptibility to pneumococcal meningitis [108].

The impact of vaccination on the pneumococcal pangenome before and after the introduction of PCV in Navajo and White Mountain Apache was investigated by Azarian and colleagues [77]. Population genetic analyses were consistent with a bottleneck effect following the introduction of PCV, followed by expansion of the non-vaccine type strains that made up the majority of the post vaccine population.

#### Pneumococcal molecular microbiology

Upper respiratory tract carriage of the pneumococcus accompanies pneumococcal disease and is the source of host-to-host transmission. Development of molecular tools and their application to assess the ‘pneumobiome’ of the upper respiratory tract in large cohorts has progressed significantly since the last WHO Pneumococcal Carriage Working Group Recommendations were published [109]. Novel approaches are being proposed with promising results. Speakers at the ISPPD-11 revealed the value of short read sequencing of extracted nucleic acid from nasopharyngeal swabs to evaluate pneumococcal antibiotic susceptibility [110] and serotype-specific carriage patterns [111], and the application of microarray to assess serotype-specific pneumococcal carriage duration and density in the first year of life [112].

Comparison of latex agglutination, whole genome sequencing and microarray for pneumococcal serotyping on nucleic acid extracted from nasopharyngeal swabs revealed high concordance for pneumococcal serotype identification between the three approaches. However, whole genome sequencing and microarray provided additional resolution of non-vaccine serotype carriage, although increasing the cost and the level of technical expertise required [113].

Identification of a new PCR target (SP2020, TIGR4 nomenclature) with 100% specificity and 99.3% positive predictive value, was revealed and suggested to be duplexed with *lyt*A for unequivocal identification of *S. pneumoniae* [114]. *Streptococcus mitis* strains that express pneumococcal serotype 1 capsule have been discovered in the oropharynx of adults using the pneumococcal *cps*1 PCR [115]. This finding has significant implications for pneumococcal serotyping approaches.

In summary, culture-independent approaches to directly interrogate the pneumobiome of the upper respiratory tract are of high value and are being actively investigated. In the near future, thorough validation of these approaches will likely lead to updated recommendations for highly sensitive detection of pneumococci in polymicrobial samples.

## Conclusions

Twenty years on from the first ISPPD held in 1998, much progress has been made to combat pneumococcal diseases globally. It was clear at the meeting that pneumococcal researchers are employing the best cutting-edge technologies to enhance our understanding of the pneumococcus and to develop new strategies to prevent, diagnose and treat pneumococcal infections.

ISPPD-11 held in 2018 gave pneumococcal researchers an opportunity to celebrate the great strides that have been made over two decades. In 2018, 58% of surviving infants had access to PCVs and 51% had been vaccinated [6].

Although there is a large burden of disease in Asia and PCV implementation has been slow, PCV introduction in several countries and the consequent impact, serve as an impetus for other countries to introduce PCVs. We acknowledge the efforts of individuals and organizations that tirelessly advocate for PCV implementation and roll-out. Another topical issue at ISPPD-11 was the persisting large burden of pneumococcal disease among the elderly. The European Commission estimates that people over 65 years old will account for a third of the population in Europe by 2050, nearly double that in 2000. Most likely, the burden of pneumococcal disease will grow with this population if nothing is done.

Other populations who continue to bear a disproportionate burden of disease and among whom urgent action is needed are refugees, internally displaced persons and the homeless. More data from refugee settings describing the burden of pneumonia by age and nutritional status are required. Millions of people in Africa’s meningitis belt remain at risk of deadly pneumococcal meningitis outbreaks despite the introduction of PCVs. Global coordination during crises with systematic evidence-based decision-making on vaccination is urgently needed. These challenges underscore the need to look beyond infant immunization programs and develop new strategies to combat disease in high-risk populations.

### ISPPD-12

ISPPD-12 will be held in Toronto, Canada from the 21st to the 25th of June 2020. This installation of ISPPD is set to have an innovative program bringing together pneumococcal researchers and partners from across the globe. More information and updates on ISPPD12 can be found at https://lp.www2.kenes.com/isppd2020/.

## Data Availability

All the symposium sessions and abstracts can be accessed at https://isppd.kenes.com/2018

## Abbreviations

ACP: All cause pneumonia
CAP: Community acquired pneumonia
CAPITA: Community-acquired Pneumonia Immunization Trial in Adults
CI: Confidence interval
CXR: Chest X-ray
DNA: Deoxyribonucleic acid
ENT: Ear, Nose and Throat
GMC: Geometric mean concentration
GSK: GlaxoSmithKline
GWAS: Genome-wide association study
HIV: Human immunodeficiency virus
IFN: Interferon
IL: Interleukin
IPD: Invasive pneumococcal disease
ISPPD: International Symposium on Pneumococci and Pneumococcal Diseases
LMIC: Low- and middle-income countries
LRI: Lower respiratory infection
NP: Nasopharyngeal
OPA: Opsonophagocytic assay
PCR: Polymerase chain reaction
PCV: Pneumococcal conjugate vaccine
PECAM: Platelet endothelial cell adhesion molecule
PERCH: Pneumonia Etiology Research for Child Health
PPV: Pneumococcal polysaccharide vaccine
RCT: Randomized control trial
RNA: Ribonucleic acid
RSV: Respiratory syncytial virus
UK: United Kingdom
USA: United States of America
VT: Vaccine type
WCW: Whole cell vaccine
WHO: World Health Organization

## References

[1] O'Brien KL, Wolfson LJ, Watt JP, Henkle E, Deloria-Knoll M, McCall N, et al. Burden of disease caused by *Streptococcus pneumoniae* in children younger than 5 years: global estimates. Lancet. 2009;374(9693):893–902. 10.1016/S0140-6736(09)61204-6 19748398

[2] Mackenzie GA, Hill PC, Jeffries DJ, Hossain I, Uchendu U, Ameh D, et al. Effect of the introduction of pneumococcal conjugate vaccination on invasive pneumococcal disease in the Gambia: a population-based surveillance study. Lancet Infect Dis. 2016. 10.1016/S1473-3099(16)00054-2 PMC4909992 26897105

[3] von Gottberg A, de Gouveia L, Tempia S, Quan V, Meiring S, von Mollendorf C (2014). Effects of vaccination on invasive pneumococcal disease in South Africa. N Engl J Med.

[4] Flasche S, Van Hoek AJ, Sheasby E, Waight P, Andrews N, Sheppard C (2011). Effect of pneumococcal conjugate vaccination on serotype-specific carriage and invasive disease in England: a cross-sectional study. PLoS Med.

[5] Estimated Hib and pneumococcal deaths for children under 5 years of age, 2000 http://www.who.int/immunization/monitoring_surveillance/burden/estimates/Pneumo_hib_2000/en/: World Health Organization; 2008 [.

[6] International Vaccine Access Center (IVAC), Johns Hopkins Bloomberg School of Public Health. VIEW-hub Global Vaccine Introduction and Implementation Report.

[7] Wahl B, O'Brien KL, Greenbaum A, Majumder A, Liu L, Chu Y, et al. Burden of *Streptococcus pneumoniae* and *Haemophilus influenzae* type b disease in children in the era of conjugate vaccines: global, regional, and national estimates for 2000-15. Lancet Glob Health. 2018;6(7):E744–E57. 10.1016/S2214-109X(18)30247-X PMC6005122 29903376

[8] Collaborators GM (2017). Global, regional, and national under-5 mortality, adult mortality, age-specific mortality, and life expectancy, 1970–2016: a systematic analysis for the Global Burden of Disease Study 2016. Lancet.

[9] Lewnard J, Givon-Lavi N, Dagan R. Early-life infection exacerbates susceptibility to otitis media. Melbourne: 11th International Symposium on Pneumococci and Pneumococcal Diseases (ISPPD-11); 2018.

[10] Binks M, Beissbarth J, Pizzutto S, Smith-Vaughan H, Andrews R, Leach A, et al. Impact of pneumococal conjugate vaccination on childhood acute lower respiratory infection in the Northern Territory of Australia: a decade of data (1996–2015). Melbourne: 11th International Symposium on Pneumococci and Pneumococcal Diseases (ISPPD-11); 2018.

[11] Agweyu A, Lilford R, English M. Clinical risk factors for mortality among children classified as non-severe pneumonia under revised World Health Organization (WHO) guidance. Melbourne: 11th International Symposium on Pneumococci and Pneumococcal Diseases (ISPPD-11); 2018.

[12] WHO. Pneumococcal Disease https://www.who.int/ith/vaccines/pneumococcal/en/2018 [Available from: https://www.who.int/ith/vaccines/pneumococcal/en/.

[13] Whitney CG, Goldblatt D, O'Brien KL (2014). Dosing schedules for pneumococcal conjugate vaccine: considerations for policy makers. Pediatr Infect Dis J.

[14] Gurung M, Kandasamy R, Thorson S, Shrestha S, Ansari I, Shah G, et al. A non-inferiority trial, comparing two-dose priming with the 10-valent pneumococcal conjugate vaccine at 6 and 10 weeks with 6 and 14 weeks in Nepali children. Melbourne: 11th International Symposium on Pneumococci and Pneumococcal Diseases (ISPPD-11); 2018.

[15] Licciardi PV, Phan T, Toh ZQ, Balloch A, Hong NVP, Trung KV, et al. Immunogenicity and memory B cell response post-booster and at 8 months of age following alternative pneumococcal vaccination strategies in Vietnam. Melbourne: 11th International Symposium on Pneumococci and Pneumococcal Diseases (ISPPD-11); 2018.

[16] Bar-Zeev N, Swarthout T, Alaerts M, Samson PW, Brown C, Phiri JC, et al. Direct and indirect impact of 13v-pneumococcal conjugate vaccine on invasive pneumococcal disease in Blantyre, Malawi. Melbourne: 11th International Symposium on Pneumococci and Pneumococcal Diseases (ISPPD-11); 2018.

[17] Tagbo B, Rowan B, Fajolu I, AbdulKadir M, Bashir M, Okunola P, et al. Paediatric bacterial meningitis surveillance in Nigeria from 2010 to 2016. Melbourne: 11th International Symposium on Pneumococci and Pneumococcal Diseases (ISPPD-11); 2018.

[18] Yang J, Siddiqui S, Uslu K, Ireland D, Windy A, Verthelyi D, et al. Characterization of pathways responsible for the weak immune response in newborns immunized with conjugate polysaccharide vaccines. Melbourne: 11th International Symposium on Pneumococci and Pneumococcal Diseases (ISPPD-11); 2018.

[19] Campbell H, Gupta S, Dolan GP, Kapadia SJ, Kumar Singh A, Andrews N (2018). Review of vaccination in pregnancy to prevent pertussis in early infancy. J Med Microbiol.

[20] Said MA, Johnson HL, Nonyane BA, Deloria-Knoll M, O'Brien KL, Team AAPBS (2013). Estimating the burden of pneumococcal pneumonia among adults: a systematic review and meta-analysis of diagnostic techniques. PLoS One.

[21] Suzuki M, Dhoubhadel BG, Ishifuji T, Yasunami M, Yaegashi M, Asoh N (2017). Serotype-specific effectiveness of 23-valent pneumococcal polysaccharide vaccine against pneumococcal pneumonia in adults aged 65 years or older: a multicentre, prospective, test-negative design study. Lancet Infect Dis.

[22] Falkenhorst G, Remschmidt C, Harder T, Hummers-Pradier E, Wichmann O, Bogdan C. Effectiveness of the 23-valent pneumococcal polysaccharide vaccine (PPV23) against pneumococcal disease in the elderly: systematic review and meta-analysis. PLoS One. 2017;12(1):e0169368. 10.1371/journal.pone.0169368 PMC5218810 28061505

[23] Naito T, Yokokawa H, Watanabe A (2018). Impact of the national routine vaccination program on 23-valent pneumococcal polysaccharide vaccine vaccination rates in elderly persons in Japan. J Infect Chemother.

[24] Suzuki M. Pneumonia in the elderly in Asia – a coming tsunami. Melbourne: 11th International Symposium on Pneumococci and Pneumococcal Diseases (ISPPD-11); 2018. 10.1186/s41479-019-0064-y PMC7001343 32042572

[25] MacIntyre R, Ridda I, Rahman B, Chughtai A, Moa A, Jones T. Long term follow-up study to examine the immunogenicity of 7-valent pneumococcal conjugate vaccine (PCV7) compared to 23-valent polysaccharide vaccine (23vPPV) in frail, hospitalized elderly. Melbourne: 11th International Symposium on Pneumococci and Pneumococcal Diseases (ISPPD-11); 2018.

[26] Bonten MJ, Huijts SM, Bolkenbaas M, Webber C, Patterson S, Gault S (2015). Polysaccharide conjugate vaccine against pneumococcal pneumonia in adults. N Engl J Med.

[27] Gessner B, Jiang Q, Werkhoven CHV, Sings H, Webber C, Scott D, et al. A public health evaluation of 13-valent pneumococcal conjugate vaccine impact on adult disease outcomes from a randomized clinical trial in the Netherlands. Melbourne: 11th International Symposium on Pneumococci and Pneumococcal Diseases (ISPPD-11); 2018.

[28] Thorrington D, van Rossum L, Knol M, de Melker H, Rumke H, Hak E (2018). Impact and cost-effectiveness of different vaccination strategies to reduce the burden of pneumococcal disease among elderly in the Netherlands. PLoS One.

[29] O'Leary ST, Maldonado YA, Kimberlin DW. Update from the advisory committee on immunization practices. J Pediatric Infect Dis Soc. 2019. 10.1093/jpids/piz058 31589289

[30] Scott A. The preventable burden of pneumonia among adults in Africa. Melbourne: 11th International Symposium on Pneumococci and Pneumococcal Diseases (ISPPD-11); 2018.

[31] Av G, Kleynhans J, Ld G, Tempia S, Meiring S, Quan V, et al. Trends in invasive pneumococcal disease among adults aged ≥25 years, South Africa, 2005–2016. Melbourne: 11th International Symposium on Pneumococci and Pneumococcal Diseases (ISPPD-11); 2018.

[32] Ludwig E, Bonanni P, Rohde G, Sayiner A, Torres A (2012). The remaining challenges of pneumococcal disease in adults. Eur Respir Rev.

[33] Jayasinghe S, Chiu C, Pennington K, Krause V, Hood J, Blyth C. Rising disparity: an increasing burden of invasive pneumococcal disease in Australian Aboriginals (2002–2014). Melbourne: 11th International Symposium on Pneumococci and Pneumococcal Diseases (ISPPD-11); 2018.

[34] Weatherholtz R, Grant L, Douglass G, Tso C, Reid R, Rudolph K, et al. Impact of pneumococcal conjugate vaccines (PCV) on invasive pneumococcal disease among American Indians living on the Navajo Reservation. Melbourne: 11th International Symposium on Pneumococci and Pneumococcal Diseases (ISPPD-11); 2018.

[35] Hammitt L. Requirements to make the switch for alternative dose vaccine schedules among indigenous peoples. Melbourne:: 11th International Symposium on Pneumococci and Pneumococcal Diseases (ISPPD-11); 2018.

[36] Kong K. Impact of PCVs on the burden of otitis in first nations populations. Melbourne: 11th International Symposium on Pneumococci and Pneumococcal Diseases (ISPPD-11); 2018.

[37] Rull M, Masson S, Peyraud N, Simonelli M, Ventura A, Dorion C (2018). The new WHO decision-making framework on vaccine use in acute humanitarian emergencies: MSF experience in Minkaman. South Sudan Confl Health.

[38] Dawood FS, Ambrose JF, Russell BP, Hawksworth AW, Winchell JM, Glass N (2011). Outbreak of pneumonia in the setting of fatal pneumococcal meningitis among US Army trainees: potential role of chlamydia pneumoniae infection. BMC Infect Dis.

[39] DeMaria A, Browne K, Berk SL, Sherwood EJ, McCabe WR (1980). An outbreak of type 1 pneumococcal pneumonia in a men's shelter. Jama.

[40] Nuorti JP, Butler JC, Crutcher JM, Guevara R, Welch D, Holder P (1998). An outbreak of multidrug-resistant pneumococcal pneumonia and bacteremia among unvaccinated nursing home residents. N Engl J Med.

[41] Tan CG, Ostrawski S, Bresnitz EA (2003). A preventable outbreak of pneumococcal pneumonia among unvaccinated nursing home residents in New Jersey during 2001. Infect Control Hosp Epidemiol.

[42] Mercat A, Nguyen J, Dautzenberg B (1991). An outbreak of pneumococcal pneumonia in two men's shelters. Chest.

[43] WHO. Accessing Affordable and Timely Supply of Vaccines for use in Humanitarian Emergencies: the Humanitarian Mechanism. https://www.who.int/immunization/programmes_systems/sustainability/The_Humanitarian_Mechanism_ToRs.pdf?ua=1: World Health Organisation; 2017.

[44] Checchi F, Margini F. Preventing pneumococcal disease in displaced populations. Melbourne: 11th International Symposium on Pneumococci and Pneumococcal Diseases (ISPPD-11); 2018.

[45] Gargano LM, Hajjeh R, Cookson ST. Pneumonia prevention: cost-effectiveness analyses of two vaccines among refugee children aged under two years, *Haemophilus influenzae* type b-containing and pneumococcal conjugate vaccines, during a humanitarian emergency, Yida camp, South Sudan. Vaccine. 2017;35(3):435–42. 10.1016/j.vaccine.2016.11.070 PMC5497221 27989625

[46] Kwambana-Adams B. Outbreaks of pneumococcal meningitis in Africa. Melbourne: 11th International Symposium on Pneumococci and Pneumococcal Diseases (ISPPD-11); 2018. 10.1186/s41479-019-0064-y PMC7001343 32042572

[47] Lessa FC, Spiller MT, Soda E, Weinberger D, Griffin MR, Grijalva CG, et al Impact of introduction of infant vaccination with 13-valent pneumococcal conjugate vaccine (PCV13) on pneumonia and invasive pneumococcal disease (IPD) hospitalizations in the United States, 2005–2014. Melbourne: 11th International Symposium on Pneumococci and Pneumococcal Diseases (ISPPD-11); 2018.

[48] Ebruke C, Roca A, Egere U, Darboe O, Hill PC, Greenwood B, et al. Temporal changes in nasopharyngeal carriage of *Streptococcus pneumoniae* serotype 1 genotypes in healthy Gambians before and after the 7-valent pneumococcal conjugate vaccine. PeerJ. 2015;3:e903. 10.7717/peerj.903 PMC4419557 25945306

[49] Kellner J, Ricketson L, Vanderkooi O, Pyra S, Tyrell G, Demczuk W, et al. Whole genome phylogenetic analysis of *Streptococcus pneumoniae* causing an outbreak of serotype 4 invasive pneumococcal disease outbreak in Alberta, Canada. Melbourne: 11th International Symposium on Pneumococci and Pneumococcal Diseases (ISPPD-11); 2018.

[50] Kwambana-Adams BA, Asiedu-Bekoe F, Sarkodie B, Afreh OK, Kuma GK, Owusu-Okyere G (2016). An outbreak of pneumococcal meningitis among older children (>/=5 years) and adults after the implementation of an infant vaccination programme with the 13-valent pneumococcal conjugate vaccine in Ghana. BMC Infect Dis.

[51] Khadka B, Amatya P, Carter MJ, Park K, Smedley M, Thorson S, et al. Comparision of WHO pneumonia case definition and radiology for the diagnosis of pneumonia in children at Patan hospital Nepal. Melbourne: 11th International Symposium on Pneumococci and Pneumococcal Diseases (ISPPD-11); 2018.

[52] Mahomed N, Philipsen R, Melendez J, Ginneken BV, Groome M, Madhi SA. Computer Aided Diagnosis for WHO Standardized Chest X-Ray Interpretation in Children, Phase 2. Melbourne: 11th International Symposium on Pneumococci and Pneumococcal Diseases (ISPPD-11); 2018.

[53] Boyd N, King C, Walker I, Zadutsa B, Bernstein M, Ahmed S, et al. Human centered design usability testing of a reusable universal pediatric pulse oximeter probe developed for children <5 years old in low-resource settings (LRS). Melbourne: 11th International Symposium on Pneumococci and Pneumococcal Diseases (ISPPD-11); 2018.

[54] Kakiuchi S, Suzuki M, Doubhadel BG, Ishifuji T, Asoh N, Ariyoshi K, et al. Detection and serotyping of pneumococci in adult pneumonia patients: Comparison of high-throughput real-time PCR assay using sputum specimens and multiplex urinary antigen detection assay. Melbourne: 11th International Symposium on Pneumococci and Pneumococcal Diseases (ISPPD-11); 2018.

[55] Lim W-S. Epidemiology of pneumococcal pneumonia in adults in the post-PCV era. Melbourne: 11th International Symposium on Pneumococci and Pneumococcal Diseases (ISPPD-11); 2018. 10.1186/s41479-019-0064-y PMC7001343 32042572

[56] Bassat Q. Minimally invasive tissue sampling to better define cause of pneumonia death. Melbourne: 11th International Symposium on Pneumococci and Pneumococcal Diseases (ISPPD-11); 2018.

[57] Sabharwal V, Yang R, Kohane D, Pelton S. Efficacy of transtympanic gemifloxacin and ciprofloxacin gel formulation against experimental otitis media in a chinchilla model due to *Streptococcus pneumoniae*. Melbourne: 11th International Symposium on Pneumococci and Pneumococcal Diseases (ISPPD-11); 2018.

[58] Hausdorff WP, Hanage WP (2016). Interim results of an ecological experiment - conjugate vaccination against the pneumococcus and serotype replacement. Hum Vaccin Immunother.

[59] Pilishvili T, Gierke R, Farley M, Schaffner W, Thomas A, Reingold A, et al. Changes in invasive pneumococcal disease (IPD) among children following 6 years of 13-valent pneumococcal conjugate vaccine (PCV13) use in the United States. 11th International Symposium on Pneumococci and Pneumococcal Diseases (ISPPD-11). Melbourne; 2018.

[60] Moore MR, Link-Gelles R, Schaffner W, Lynfield R, Lexau C, Bennett NM (2015). Effect of use of 13-valent pneumococcal conjugate vaccine in children on invasive pneumococcal disease in children and adults in the USA: analysis of multisite, population-based surveillance. Lancet Infect Dis.

[61] Savulescu C, Colzani E, Pastore-Celentano L, Hanquet G, group S. Impact of higher-valency pneumococcal conjugate vaccines on invasive pneumococcal disease in children under 5 years (2011–2016): SpIDnet– a European multicentre study. Melbourne: 11th International Symposium on Pneumococci and Pneumococcal Diseases (ISPPD-11); 2018.

[62] Ladhani S, Collins S, Djennad A, Sheppard C, Borrow R, Fry N, et al. Rapid increase in non-vaccine serotypes causing invasive pneumococcal disease in England and Wales: a prospective national observational cohort study, 2000–2017. Melbourne: 11th International Symposium on Pneumococci and Pneumococcal Diseases (ISPPD-11); 2018. 10.1016/S1473-3099(18)30052-5 29395999

[63] Lewnard JA, Hanage WP. Making sense of differences in pneumococcal serotype replacement. Lancet Infect Dis. 2019. 10.1016/S1473-3099(18)30660-1 30709666

[64] Senghore M, Kwambana-Adams B, Worwui A, Teintcheu PE, Salaudeen R, Av G, et al. Evolution of pneumococci causing invasive disease in the Gambia before and after the introduction of pneumococcal conjugate vaccines. Melbourne: 11th International Symposium on Pneumococci and Pneumococcal Diseases (ISPPD-11); 2018.

[65] Mungall B, Soumahoro L, Hoet B. Correlating PCV impact on carriage with impact on disease: a systematic review. Melbourne: 11th International Symposium on Pneumococci and Pneumococcal Diseases (ISPPD-11); 2018.

[66] Tuivaga E, Reyburn R, Nguyen C, Ratu T, Nand D, Kado J, et al. Temporal association of 10-valent pneumococcal conjugate vaccine introduction with all-cause, severe, hypoxic and radiological pneumonia hospitalisations in children and elderly in Fiji: a time-series analysis. Melbourne: 11th International Symposium on Pneumococci and Pneumococcal Diseases (ISPPD-11); 2018.

[67] Shrestha S, Thorson S, Gurung M, Carter MJ, Kandasamy R, Garcia C, et al. A comprehensive pneumococcal vaccine impact assessment in Nepal. Melbourne: 11th International Symposium on Pneumococci and Pneumococcal Diseases (ISPPD-11); 2018.

[68] Ravikumar KL, Govindan V, Nagaraj G. What is happening with PCV introduction in India?. Melbourne: 11th international Symposium on Pneumococci and Pneumococcal Diseases (ISPPD-11); 2018.

[69] Chan J, Mollendorf CV, Dunne E, Fox K, Hinds J, Vincente SL, et al. Using nasopharyngeal carriage surveillance in children hospitalised with pneumonia to determine the pneumococcal conjugate vaccine coverage required for indirect immunity in Mongolia. Melbourne: 11th International Symposium on Pneumococci and Pneumococcal Diseases (ISPPD-11); 2018.

[70] Croucher N, Corander J, Colijn C. Optimising vaccine formulations to minimise disease and antibiotic treatment failures. Melbourne: 11th International Symposium on Pneumococci and Pneumococcal Diseases (ISPPD-11); 2018.

[71] Ojal J, Griffiths U, Hammitt LL, Adetifa I, Akec D, Tabu C, et al. The merits of sustaining pneumococcal vaccination after transitioning from GAVI support – a cost-effectiveness study for Kenya. Melbourne: 11th International Symposium on Pneumococci and Pneumococcal Diseases (ISPPD-11); 2018.

[72] Sbarra AN, Shioda K, Warren JL, Oliveira LHD, Weinberger DM. Improving credibility of PCV impact estimates using pooled analysis. Melbourne: 11th International Symposium on Pneumococci and Pneumococcal diseases (ISPPD-11); 2018.

[73] Choi YH, Miller E. Modelling the impact of changing from a 2+1 to 1+1 PCV13 schedule in England and Wales. Melbourne: 11th International Symposium on Pneumococci and Pneumococcal Diseases (ISPPD-11); 2018.

[74] Rupp R, Johnston W, Hurley D, Petrecz M, Li J, Nolan K, et al. A dose ranging study of 15-valent pneumococcal conjugate vaccine (PCV-15) in healthy infants. Melbourne: 11th International Symposium on Pneumococci and Pneumococcal Diseases (ISPPD-11); 2018.

[75] Skinner J, Kaufhold R, McGuinness D, McHugh P, Winters M, Smith W, et al. Immunogenicity of PCV24, a next generation pneumococcal conjugate vaccine, in Macaques. Melbourne: 11th International Symposium on Pneumococci and Pneumococcal Diseases (ISPPD-11); 2018.

[76] Lalwani S, Bavdekar A, Ramanan PV, Dhere R, Sethna V. Safety and immunogenicity of a 10-valent pneumococcal conjugate vaccine in healthy PCV-naïve Indian toddlers – a phase 2 double-blind randomized controlled trial. Melbourne: 11th International Symposium on Pneumococci and Pneumococcal Diseases (ISPPD-11); 2018.

[77] Stacey H, Rosen J, Peterson J, Williams-Diaz A, Gakhar V, Sterling T, et al. Safety and immunogenicity of 15-valent pneumococcal conjugate vaccine (PCV-15) compared to PCV-13 in healthy older adults. Melbourne: 11th International Symposium on Pneumococci and Pneumococcal Diseases (ISPPD-11); 2018. 10.1080/21645515.2018.1532249 PMC6605726 30648919

[78] Goulart C, Rodriguez D, Kanno AI, Lu YJ, Malley R, Leite LC (2017). Recombinant BCG expressing a PspA-PdT fusion protein protects mice against pneumococcal lethal challenge in a prime-boost strategy. Vaccine.

[79] Ren B, Li J, Genschmer K, Hollingshead SK, Briles DE (2012). The absence of PspA or presence of antibody to PspA facilitates the complement-dependent phagocytosis of pneumococci in vitro. Clin Vaccine Immunol.

[80] Khan MN, Sharma SK, Filkins LM, Pichichero ME. PcpA of *Streptococcus pneumoniae* mediates adherence to nasopharyngeal and lung epithelial cells and elicits functional antibodies in humans. Microbes Infect. 2012;14(12):1102–10. 10.1016/j.micinf.2012.06.007 PMC3490615 22796387

[81] Bologa M, Kamtchoua T, Hopfer R, Sheng X, Hicks B, Bixler G (2012). Safety and immunogenicity of pneumococcal protein vaccine candidates: monovalent choline-binding protein a (PcpA) vaccine and bivalent PcpA–pneumococcal histidine triad protein D vaccine. Vaccine.

[82] Rahman T, Thornton R, Corscadden K, Granland C, Francis J, Solomon V, et al. Antibody titres against pneumococcal protein vaccine antigens in Papua New Guinean children at high risk of pneumococcal disease. Melbourne: 11th International Symposium on Pneumococci and Pneumococcal Diseases (ISPPD-11); 2018.

[83] Novick S, Shagan M, Blau K, Lifshitz S, Givon-Lavi N, Grossman N, et al. Adhesion and invasion of *Streptococcus pneumoniae* to primary and secondary respiratory epithelial cells. Mol Med Rep. 2017;15(1):65–74. 10.3892/mmr.2016.5996 PMC5355668 27922699

[84] Vernatter J, Pirofski LA (2013). Current concepts in host-microbe interaction leading to pneumococcal pneumonia. Curr Opin Infect Dis.

[85] Aprianto R, Slager J, Holsappel S, Veening JW (2016). Time-resolved dual RNA-seq reveals extensive rewiring of lung epithelial and pneumococcal transcriptomes during early infection. Genome Biol.

[86] Jhelum H, Sori H, Sehgal D. A novel extracellular vesicle-associated endodeoxyribonuclease helps *Streptococcus pneumoniae* evade neutrophil extracellular traps and is required for full virulence. Sci Rep. 2018;8(1):7985. 10.1038/s41598-018-25865-z PMC5964101 29789571

[87] Panagiotou S, Khandaker S, Chaguza C, Yangl M, Kadioglu A. Further understanding of pathogenesis and host immunity in pneumococcal meningitis. Melbourne: 11th International Symposium on Pneumococci and Pneumococcal Diseases (ISPPD-11); 2018

[88] Iovino F, Engelen-Lee JY, Brouwer M, van de Beek D, van der Ende A, Valls Seron M (2017). pIgR and PECAM-1 bind to pneumococcal adhesins RrgA and PspC mediating bacterial brain invasion. J Exp Med.

[89] Iovino F, Thorsdottir S, Henriques-Normark B. Receptor Blockade: A Novel Approach to Protect the Brain From Pneumococcal Invasion. J Infect Dis. 2018. 10.1093/infdis/jiy193 29701809

[90] Campo JJ, Turner P, Le TQ, Liang X, Croucher NJ, Goldblatt D. Proteomic antibody profiles during the first two years of life in infants on the Thailand-Myanmar border. Melbourne: 11th International Symposium on Pneumococci and Pneumococcal Diseases (ISPPD-11); 2018.

[91] Croucher N, Le T, Pablo J, Hung C, Teng A, Tate A, et al. Panproteome-wide view of the immunological response to a whole cell pneumococcal vaccine. Melbourne: 11th International Symposium on Pneumococci and Pneumococcal Diseases (ISPPD-11); 2018.

[92] Mitsi E, Carniel B, Jochems SP, Rylance J, Reine J, Schanoski AS, et al. Cross-talk of alveolar macrophages and T-cells boosts the lung immunity post nasopharyngeal pneumococcal colonisation. Melbourne: 11th International Symposium on Pneumococci and Pneumococcal Diseases (ISPPD-11); 2018.

[93] Abudulai L, Fernandez S, Corscadden K, Kirkham LA, Hunter M, Post JJ, et al. Production of IgG2 antibodies to pneumococcal polysaccharides requires ICOS+ circulating memory follicular helper T-cells and is impaired by HIV-1 infection. Melbourne: 11th International Symposium on Pneumococci and Pneumococcal Diseases (ISPPD-11); 2018.

[94] Makenga G, Mtove G, Yin K, Booy R. Immune response to pneumococcal conjugate vaccine (PCV13) serotypes in Tanzanian children with HIV/AIDS. Melbourne: 11th International Symposium on Pneumococci and Pneumococcal Diseases (ISPPD-11); 2018.

[95] Apiwattanakul N, Thomas PG, Kuhn RE, Herbert DR, McCullers JA (2014). Helminth infections predispose mice to pneumococcal pneumonia but not to other pneumonic pathogens. Med Microbiol Immunol.

[96] McCullers JA (2014). The co-pathogenesis of influenza viruses with bacteria in the lung. Nat Rev Microbiol.

[97] Toizumi M, Nguyen HA, Iwasaki C, Vo MH, Takegata M, Kitamura N, et al. *Streptococcus pneumoniae* interacts with respiratory syncytial virus and increases severity of respiratory illness among Vietnamese children. Melbourne: 11th International Symposium on Pneumococci and Pneumococcal Diseases (ISPPD-11); 2018.

[98] Khandaker S, Yang M, Kadioglu A. Effect of respiratory syncytial virus infection on pneumococcal colonisation and invasive disease. Melbourne: 11th International Symposium on Pneumococci and Pneumococcal Diseases (ISPPD-11); 2018.

[99] Law A, Shears R, Grencis R, Kadioglu A, Neill D. Low dose *Trichuris muris *infection impairs host immune control of *Streptococcus pneumoniae* nasopharyngeal carriage. Melbourne: 11th International Symposium on Pneumococci and Pneumococcal Diseases (ISPPD-11); 2018.

[100] Smith AM, Adler FR, Ribeiro RM, Gutenkunst RN, McAuley JL, McCullers JA, et al. Kinetics of coinfection with influenza A virus and *Streptococcus pneumoniae*. Plos Pathog. 2013;9(3). 10.1371/journal.ppat.1003238 PMC3605146 23555251

[101] Ghoneim HE, McCullers JA (2014). Adjunctive corticosteroid therapy improves lung immunopathology and survival during severe secondary pneumococcal pneumonia in mice. J Infect Dis.

[102] Califano D, Furuya Y, Metzger DW (2018). Effects of influenza on alveolar macrophage viability are dependent on mouse genetic strain. J Immunol.

[103] Lewnard J, Givon-Lavi N, Dagan R. Pneumococcal serotype-specific interaction with non-typeable *Haemophilus influenzae* (NTHI) is consistent in acute bacterial conjunctivitis and otitis media. Melbourne: 11th International Symposium on Pneumococci and Pneumococcal Diseases (ISPPD-11); 2018.

[104] Hiller L. Genomics plasticity: tissue tropism, gene regulation and strain-strain interactions. Melbourne: 11th International Symposium on Pneumococci and Pneumococcal Diseases (ISPPD-11); 2018.

[105] Lehtinen S. The role of duration of carriage in the dynamics of drug and multi-drug resistance. Melbourne: 11th International Symposium on Pneumococci and Pneumococcal Diseases (ISPPD-11); 2018.

[106] Gladstone R, Md P, Wolter N, Madhi S, Nzenze S, Lo S, et al. Beyond serotype: the contribution of genotype to invasiveness. Melbourne: 11th International Symposium on Pneumococci and Pneumococcal Diseases (ISPPD-11); 2018.

[107] Ebruke C, Senghore M, Worwui A, Tientcheu PE, Okoi C, Salaudeen R, et al. Comparative genomic analysis of *Streptococcus pneumoniae* Serotype 1 strains from West Africa (1996–2016). Melbourne: 11th International Symposium on Pneumococci and Pneumococcal Diseases (ISPPD-11); 2018.

[108] Lees J, Ferwerda B, Kremer P, Wheeler N, Mercedes VS, Croucher N, et al. Joint sequencing of host and pathogen genomes reveals the genetics underlying susceptibility to and severity of pneumococcal meningitis. Melbourne: 11th International Symposium on Pneumococci and Pneumococcal Diseases (ISPPD-11); 2018.

[109] Satzke C, Turner P, Virolainen-Julkunen A, Adrian PV, Antonio M, Hare KM, et al. Standard method for detecting upper respiratory carriage of *Streptococcus pneumoniae*: updated recommendations from the World Health Organization pneumococcal carriage working group. Vaccine. 2013;32(1):165–79. 10.1016/j.vaccine.2013.08.062 24331112

[110] Manenzhe R, Moodley C, Dube FS, Wright M, Lennard K, Zar H, et al. Shotgun sequencing to elucidate pneumococcal strain level nasopharyngeal colonization patterns and antimicrobial resistance in a South African birth cohort. Melbourne: 11th international symposium on pneumococci and pneumococcal diseases (ISPPD-11).

[111] Dube FS, BS D, Nduru P, Gladstone R, Zar HJ, Nicol MP. Characterisation of lineage and locus-specific variations associated with pneumococcal carriage dynamics post PCV-13 implementation in an African birth cohort: the Drakenstein Child Health Study. Melbourne: 11th International Symp​o​sium​​ on Pneumococci and Pneumococcal Diseases (ISPPD-11); 2018.

[112] Dunne E, Murad C, Sudigdoadi S, Fadlyana E, Tarigan R, Pell C, et al. Pneumococcal carriage dynamics during the first year of life: A longitudinal study in Indonesian infants. Melbourne: 11th International Symposium on Pneumococci and Pneumococcal Diseases (ISPPD-11); 2018.

[113] Swarthout TD, Gori A, Bar-Zeev N, Everett D, Kamngona AW, Brown C, et al. Pneumococcal serotyping of Malawi carriage samples by latex agglutination, whole genome sequencing (PneumoCat) and DNA microarray is highly concordant: “which should you choose?” Melbourne: 11th international symposium on pneumococci and pneumococcal diseases (ISPPD-11); 2018.

[114] Handem S, Tavares DA, Carvalho RJ, Hd L, Hinds J, Sá-Leão R. Identification of *Streptococcus pneumoniae* by a real-time PCR assay targeting SP2020. Melbourne: 11th International Symposium on Pneumococci and Pneumococcal Diseases (ISPPD-11); 2018.

[115] Lessa FC, Beall B, Milucky J, Rouphael N, Bennett NM, Talbot HK, et al. Editors. *Streptocccus mitis* expressing pneumococcal serotype 1 capsule. Melbourne: 11th international symposium on pneumococci and pneumococcal diseases (ISPPD-11); 2018. 10.1038/s41598-018-35921-3 PMC6299277 30568178

